# A Few Practical Observations on the Treatment of the Pulp

**Published:** 1883-09

**Authors:** W. E. Harding

**Affiliations:** England


### A Few Practical Observations on the Treatment of the Pulp.

BY W. E. HARDING, L.D.S., ENGLAND.

Read at the Annual Meeting of the Midland Branch held at Shrewsbury, April 25th, 1883.

The subject of pulp treatment is one on which there is great divergence of opinion, and as it is one of every day occurrence it requires all our skill and patience. In the "good old days," the treatment of an exposed pulp was the extraction of the tooth, but that mode of precedure has long been exploded by the progress of Dental Science.

The first question the dental surgeon of to-day asks himself is not—Can I save the tooth, but can I save the pulp? This, gentlemen, has added much to our labor and anxieties, but it has also contributed much to our professional usefulness to mankind.

We all know how easily and successfully cases of accidental exposure can be treated. My usual plan is to swab out the cavity with carbolic acid, which will arrest the bleeding, then place over the point of exposure a small piece of court plaster or blotting paper coated with carbolized resin, and cover this with a layer of Fletcher's artificial dentine. This can be applied in a more plastic state than the phosphate fillings, and unlike oxychloride of zinc it is non-irritant and easily removed if necessary. When this has hardened the permanent filling may be proceeded with at the same sitting.

The reason these cases are so easily dealt with is that the pulp is quite healthy, and if covered with a non-conductor *at once*, so as to exclude the germs of micro-organisms, it will heal by first intention.

But in those cases where the exposure results from caries the difficulties are vastly greater, as the pulp is almost sure to be inflamed, and probably suppurating. My experience of these cases treated by capping is the reverse of satisfactory, the percentage of successful cases being small, and on this point I hope some of the gentlemen present will give us the benefit of their experience.

The first point to determine is the condition of the pulp. The history of the case is often deceptive, for if the cavity is in such a position as not to be exposed to the impact of food it may not have given much pain, and yet be suppurating; but in the odor we have a certain means of diagnosis. If that peculiar phosphatic odor is present, I very much doubt the possibility of saving the pulp, and think any attempt at capping will result in its death, and eventually in a chronic abscess; in such cases the destruction of the pulp is, I think, by far the best treatment.

Should capping be determined upon, the application to the exposed surface of iodoform and eucalyptus oil has generally proved the most successful. It may then be covered with artificial dentine—and in these cases it is always safer to put in a temporary filling for a few weeks, which can be removed in case of pain. Hill's gutta percha over the artificial dentine answers very well.

In the destruction of the pulp the points to be considered are: 1. The destroying agent. 2. The removal of the dead pulp. 3. The treatment of the canals. 4. The filling of the canals and pulp chamber.

Arsenious acid is the common agent used to destroy the pulp. I

have found Baldock's preparation very good; or the arsenic, which should be rubbed to an impalpable powder, may be mixed into a paste with oil of cloves, *not* carbolic acid, as that forms an eschar which retards the action of the arsenic; where possible it is best not to cover it with sandarach or mastic, but with Hill's gutta percha or even wax, as the varnish is apt to coat the pulp and prevent the action of the arsenic—being careful to avoid pressure on the exposed pulp or considerable pain will be the result.

Before applying the arsenic it is very important to get easy access to the cavity. Thus if the cavity be in the distal surface of one of the molars it must be freely opened into the crown with enamel chisels, and the pulp must then be thoroughly exposed. The neglect of this is one of the most fruitful sources of pain, for the drug irritates the pulp without destroying it. The arsenic may remain in the tooth for forty-eight hours, or even a week, when, if the pulp is dead, it should be at once removed with a barbed extractor; though some practitioners recommend the application of a solution of tannin, believing that it makes the dead tissue tough and therefore easier to remove.

The next stage is the treatment of the canals, and this must depend very much upon the state of the pulp before it was destroyed. If the body of the pulp is not decomposed the canals may be filled at once, but should decomposition have extended much beyond the surface of the pulp, the canals will require further treatment to destroy the micro-organisms which are the agents of decomposition. Mr. Chas. Tomes' suggestion to apply the rubber-dam and to remove the pulp, while the tooth is inundated with eucalyptus oil and so prevent the entrance of these micro-organisms—apart from the difficulties of manipulation—has a great deal to recommend it and would be truly following out Professor Lister's theory of antiseptic treatment, viz., to prevent the entrance to the wound of the germs which may be floating in the air.

We have lately had many new antiseptics proposed for dressing the pulpless canals. Prominent amongst them stand eucalyptus oil and iodoform, both are very valuable, but our old friends creasote and carbolic acid are not by any means to be neglected. The strong and persistent odor of both eucalyptus and iodoform is a great objection to their use, as it quite overpowers any odor from the canals, and so prevents your being certain when you have got them into a healthy condition. I must prefer a solution of iodine and carbolic acid in spirit, ʒi of the crystals of each to ʒii of rectified spirit. The rapid action of iodine on steel instruments being the greatest objection to its use, this may be obviated by using platinum broaches.

The canals being thoroughly disinfected, the next consideration is the material with which they are to be filled. The difficulties encountered in the manipulation of gold almost preclude its use, especially as we have a far better material in oxychloride of zinc. Some shreds of cotton wool wrapped round one of Donaldson's bristles should be saturated with the oxychloride—mixed rather thin—and then carried quite up to the end of the canal, the bristle being withdrawn with a kind of up-and-down pumping movement.

Carbolic acid may be used instead of oxychloride with excellent results. I have in my own mouth a central incisor which was treated for alveolar abscess by Dr. Mordaunt Stevens, the canal being filled with creasote and wool, the cavity with gold, it has now been quite comfortable for twelve years, and has no fistulous opening.

It is always wiser to put in for a few weeks a temporary filling of gutta percha, which, in case of trouble, can be easily removed.

In cases where there is a fistulous opening on the gum connected with the root of the tooth, it is important that the antiseptic agent used should be pumped through the canal and out on to the gum; for this purpose Farrar's Abscess Syringe will be found useful. But after all our care and perseverance there will remain a number of cases which seem most intractable, generally cases in which the pulp has long been dead. The only treatment for these seems to be rhisodontrophy.

The rubber dam will be found very valuable when dressing the canals, as it not only keeps out the saliva, but protects the patient's lips from the caustic action of the agents used.

In conclusion, the importance of thoroughness in every detail cannot be too strongly urged.—*Journal of the British Dental Asso.*

